# Inbox message prioritization and management approaches in primary care

**DOI:** 10.1093/jamiaopen/ooae135

**Published:** 2024-11-11

**Authors:** Nate C Apathy, Katelyn Hicks, Lucy Bocknek, Garrett Zabala, Katharine Adams, Kylie M Gomes, Tara Saggar

**Affiliations:** Health Policy & Management, University of Maryland School of Public Health, College Park, MD 20742, United States; Regenstrief Institute, Indianapolis, IN 46202, United States; Georgetown University School of Medicine, Washington, DC 20007, United States; MedStar Health National Center for Human Factors in Healthcare, MedStar Health Research Institute, Columbia, MD 21044, United States; MedStar Health National Center for Human Factors in Healthcare, MedStar Health Research Institute, Columbia, MD 21044, United States; MedStar Health Center for Biomedical Informatics and Data Science, MedStar Health Research Institute, Columbia, MD 21044, United States; MedStar Health National Center for Human Factors in Healthcare, MedStar Health Research Institute, Columbia, MD 21044, United States; MedStar Health, Columbia, MD 21044, United States

**Keywords:** patient messaging, physician inbox, physician burnout, qualitative research

## Abstract

**Objectives:**

Patient messaging to clinicians has dramatically increased since the pandemic, leading to informatics efforts to categorize incoming messages. We examined how message prioritization (as distinct from categorization) occurs in primary care, and how primary care clinicians managed their inbox workflows.

**Materials and Methods:**

Semi-structured interviews and inbox work observations with 11 primary care clinicians at MedStar Health. We analyzed interview and observation transcripts and identified themes and subthemes related to prioritization and inbox workflows.

**Results:**

Clinicians widely reported that they did not prioritize messages due to time constraints and the necessity of attending to all messages, which made any prioritization purely additive to overall inbox time. We identified 6 themes and 14 subthemes related to managing inbox workloads. The top themes were (1) establishing workflow norms with different teams, primarily medical assistants (MAs); (2) boundary-setting with patients, other clinicians, and with themselves; and (3) message classification heuristics that allowed clinicians to mentally categorize messages that required follow-up, messages that could be quickly deleted or acknowledged, and purely informational messages that ranged in clinical utility from tedious to valuable for care coordination.

**Discussion:**

Time constraints in primary care prevent clinicians from prioritizing their inbox messages for increased efficiency. Involvement of MAs and co-located staff was successful; however, standardization was needed for messaging workflows that involved centralized resources like call centers. Organizations should consider ways in which they can support the establishment and maintenance of boundaries, to avoid this responsibility falling entirely on clinicians.

**Conclusion:**

Clinicians generally lack the time to prioritize patient messages. Future research should explore the efficacy of collaborative inbox workflows for time-savings and management of patient messages.

## Background and significance

In the wake of the Coronavirus disease pandemic (COVID-19), patient portal messaging to physicians swelled 157% compared to pre-pandemic levels and since then has remained a significant source of physician burden.^1^ This is due in part to an overall increase in portal message volume and a wide variation in message content ranging from routine appointment requests or medication refills to complex description of symptoms.^2^ This trend is likely to continue due to an increasing shift to asynchronous care and patient familiarity with virtual care modalities driven by the pandemic.^1^^,^^3^^,^^4^ These factors, coupled with a widespread adoption of utilizing electronic health records (EHRs) prior to the pandemic,^5^ have effectively solidified patient expectations of future communication with their physician through a portal.

Despite the increased adoption of patient portals^6^ and the challenges that come with them, few solutions have been developed to help clinical teams deal with the additional work derived from patient messaging. Some organizations have established different workflows and strategies for inbox management, including “message pools” within clinical teams that allow multiple users to “share” an inbox, thus allowing any of the team members to handle a given message.^7^^,^^8^ However, team-based messaging still requires someone on the team to complete the taxing cognitive task of reviewing a message and assessing its urgency and acuity for prioritization. This need for improved prioritization and message triage has fostered a growing literature focused on classification algorithms to tag messages according to broad categories of content like “logistics” or “reporting symptoms.”^9–14^ However, these approaches to *categorize* do little to facilitate *prioritization* of messages—especially within categories—to ensure that high-priority messages are addressed quickly and each team member is handling the messages best matched with their expertise and role. While abstraction of message content into categories may effectively “interpret” or “read” a message, the same abstraction removes important clinical details that inform prioritization, which is a key cognitive task and a core contributor to the inbox burden that physicians experience. For example, classifying a message as “logistics” (a common categorization across several studies)^9^^,^^11^ lumps together messages that may be requesting an urgent appointment for an acute exacerbation of illness with messages asking for a future appointment to be canceled or informing the clinical team that the patient will be 10 min late. These 3 “logistics” messages have distinct priorities that can be masked by categorization algorithms. Little is known regarding how message content can be leveraged to facilitate prioritization to save physicians’ time, cognitive load, and stress. In particular, the clinical cognition supporting message prioritization—rather than categorization—remains largely unexplored. We aimed to address this knowledge gap by studying the specific aspects of messages that physicians use to prioritize (and de-prioritize) patient messages, focusing on primary care as the specialty that has the greatest overall volume of patient messages and substantial use of team-based care models.^15^

### Objective

The purpose of our study was to examine how message prioritization (as distinct from categorization) occurs in primary care. Our goal was to identify generalizable message prioritization practices that could be operationalized into informatics tools (eg, algorithms, decision support tools, natural language processing models, etc.) to support automated prioritization of patient messages upstream of physician inboxes. Our findings have implications for the creation and sharing of prioritization concepts that could be used by organizations to refine and create tailored prioritization algorithms to support care teams’ inbox management. Conversely, it is possible that the time constraints of primary care hinder physicians’ ability to prioritize at all given the deluge of messages and other pressures. In that case, our findings can inform best practices for optimizing message management workflows within primary care teams.

## Materials and methods

### Study design

Our study leveraged semi-structured interviews and direct observations to elicit the elements of patient messages that primary care providers (PCPs) use to prioritize and de-prioritize messages (“prioritization concepts”). We used email distribution across clinicians in the MedStar Health primary care service line to recruit PCPs for interviews and observations, and early participants also provided referral-based sampling to reach out to additional individuals for recruitment. During the recruitment process, the study team examined each potential participant’s recent volume of inbox messages, both directly from patients and from other sources, to ensure the recruitment of participants with sufficient inbox message “exposure” to represent PCPs with nontrivial inbox burden. The interviewers had no prior relationships with the participants. Interviews and observations took place between June 2023 and October 2023. Our inclusion criteria required PCPs to carry their own panel of patients and use the Oracle Cerner EHR inbox for communication with patients and the clinical team. All PCP participants gave informed consent to be interviewed and observed. No patients were recruited for the study, as our interviews and observations took place outside of the context of patient visits.

### Data collection and analysis

We conducted semi-structured interviews and inbox work observations with participating PCPs. Interviews lasted between 45 and 60 min and featured topics such as inbox message prioritization; preferred workflow(s) for answering messages; and use of team-based strategies to manage clinician inbox volume. Interviews had 2 distinct phases. In the first phase, we asked PCPs questions about the demographics of their patient populations, their estimated inbox message volume, prioritization strategies, and team-based inbox management strategies in place at their practice. This phase was used to understand how PCPs perceived their inbox volume and various management strategies outside of the context of a specific message.

The second phase of our interviews consisted of direct observation of PCP inbox work. During this phase, PCPs opened their EHR inbox (Oracle Cerner’s Message Center), shared their screen, and addressed real messages according to their own preferred workflows in the live environment. During observations, the study team prompted PCPs to verbalize their cognitive processes in managing or responding to messages to both illustrate their thinking and cue interviewer follow-up questions. As inbox triage work proceeded, the study team also asked questions specific to prioritization if the PCP did not verbalize a specific prioritization concept during a given message action (eg, “What about that message led you to delete it without responding or follow-up?”).

All interviews and observations were conducted via the Microsoft Teams platform, recorded, and transcribed for thematic analysis. Interviews were initially auto-transcribed using the MS Teams automated transcription service. The study team reviewed transcripts for accuracy and completeness, then coded the transcripts to identify inbox prioritization and work management themes using Microsoft Excel.

Two study team members (N.C.A. and K.H.) used a grounded theory approach for codebook development. We reviewed early interview transcripts to establish an initial set of thematic codes (themes and subthemes) that described the inbox management strategies and prioritization concepts that participants noted. The code list was iteratively developed as additional themes and subthemes became apparent through further interviews, with continued iterations until thematic saturation was achieved. All interview transcripts were initially coded by one team member to associate PCP responses with specific subthemes; this initial code was reviewed for agreement by a second team member. Any coding discrepancies were resolved via consensus with a third study team member.

To analyze coded transcripts, we summarized the most prevalent themes and subthemes and captured illustrative quotes from transcripts. For themes representing binary constructs (eg, positive sentiment toward the clinician inbox), we calculated the proportion of participants that expressed either value of the construct. We present below summary statistics of our sample as well as the most common themes and subthemes accompanied by illustrative quotes.

## Results

We conducted semi-structured interviews and observations with 11 primary care clinicians (10 female and 1 male). Three clinicians were nurse practitioners managing their own patient panel and the remainder were physicians. Clinicians ranged in tenure at MedStar Health from 30 years to less than 9 months.

### Prioritization of inbox messages

The EHR messaging interface included a “Priority Items” section to which team members could route high-priority or urgent messages (eg, critical laboratory values), which most clinicians indicated they would typically review first, if there were any messages flagged as such. It was not possible for patients to send messages directly to this section of the inbox from the portal. Six clinicians reported that they did not do any prioritization of inbox messages within other inbox sections, citing reasons largely centering around time constraints in the primary care ecosystem. Clinicians reported that they rarely had time to holistically assess the inbox to prioritize patient messages that required relatively more urgent attention, and since all messages would have to be handled eventually, simply going from top to bottom (or bottom to top) was the preferred strategy.

***As they come in, I just do them.*** *I go in order and if it’s something that I can come back to, that’s something I’ll look at and leave them right there.* (Participant 4)

***I’m very top to bottom …*** *I definitely am, you know,****just clear it out top to bottom*** *because it keeps me on track.* (Participant 5)

When clinicians mentioned workflows that involved sequencing of different message review tasks, it was in reference to navigating different sections of the Oracle Cerner Message Center. For example, one clinician noted that “*I don’t have any particular sequence, but I’m going to go through documents first, then results, then [patient] messages …*” referring to the different navigation sections of the Message Center interface.

### Inbox management approaches

The most common themes in our discussions of inbox management approaches were (1) workflow norms and rules with clinical and administrative teams (*n* = 11); (2) boundary-setting with other clinicians, patients, and with themselves (*n* = 10); and (3) message classification heuristics describing how clinicians thought about different types of patient messages (*n* = 9). Primary themes, subthemes, and representative quotes are presented in Table 1, and secondary themes and subthemes are included in the Appendix.

**Table 1.** Summary of primary themes and subthemes.

Theme	Subtheme	Summary of findings	Illustrative quote
Inbox prioritization	Lack of message prioritization	Participants reported that they did not explicitly prioritize messages, primarily due to time constraints.	“As they come in, I just do them. I go in order and if it’s something that I can come back to, that’s something I’ll look at and leave them right there.” (Participant 4)
Workflow norms and rules	Team-based workflows with clinical teams	Medical assistants (MAs) were the clinical care team members most likely to be involved in inbox message management approaches.	Yeah, like for example, when I’m addressing my labs, I send my lab results to my medical assistant. Now if there is something that my medical assistant is getting a glitch or patient is asking more questions, she will route that message back to me. (Participant 10)
	Team-based workflows with administrative teams	Participants reported that management of messages with administrative teams tended to involve more back-and-forth communication and were often discussed as a source of friction.	We have a clinical pool and an administrative pool. The administrative pool will pick up the messages that are for patients needing to get scheduled and they’ll try to put those [on the schedule]. But then sometimes that will come back, because if there’s no availability, then they send us a message (Participant 4)
	Clinical scope of practice and message routing	The Connected Care Program at MedStar Health provides virtual NP support to physicians. This program was mentioned favorably on several occasions as a valuable team-based workflow for medication refills.	Connected Primary Care where now I have a group that’s refilling my medications, except for the controlled substance ones or ones that are questionable. So that’s been like 2 weeks, it’s significantly decreased messages. (Participant 1)
	Workflows and habits for when to reply to messages	Participants expressed consistently checking their message inbox throughout the clinic day, primarily to avoid reaching the end of the day with a large amount of unread messages in their inbox.	I’m constantly looking at [my messages] very briefly during the day, so I don’t end up at 4 o’clock with 20 messages. (Participant 1)
Boundary setting	Boundary-setting with patients	Participants noted the necessity of proactively communicating with patients about expectations for the use of the portal and clinician messaging capabilities.	I have a smart text when they [first] come in … saying “Results will be in the inbox. Give me a little bit of time to review it. Don’t put anything urgent in the inbox.” (Participant 11)
	Boundary-setting with other clinicians	The most common use case for establishing boundaries with other clinicians was related to covering other clinicians’ inboxes during their vacations. Participants noted varied cultural expectations across clinics.	“Something that is important to know is that I have created an office as the medical director where when someone’s out the other doctors really try to make them come back to zero messages… It is a group mission and what goes around comes around.” (Participant 1)
	Boundary-setting with oneself	Most participants who mentioned self-imposed boundaries on inbox management illustrated a belief that establishing sustainable work patterns for managing the inbox fell entirely on themselves, with little indication of organizational supports.	So just kind of drawing that line… that’s something I definitely need to work on because that will give me a better work-life balance and decrease some of these messages. (Participant 4)
Message classification heuristics	Messages requiring follow-up or additional chart review	While these messages were relatively rare, clinicians indicated that these messages required a disproportionate amount of time and cognitive energy.	That one would probably be something I would say like, OK, I need to look into it further cause I need to look at her chart. I need to figure out what other kind of medical conditions she has… I was going to look into that, but just given time, I’m gonna probably skip ahead right now and come back to that one. (Participant 5)
	Messages requiring minimal work	A large share of messages fell into the category of messages that could be quickly managed via immediate deletion or acknowledgement.	So quick [messages like] “I need an order for this.” I’ll put that in real quick. [Or a message asking] “Do you think the patient needs to be seen for this issue?” I reply back, “Yes, schedule the patient for an urgent appointment.” It’s a quick yes, no or a couple clicks. (Participant 4)
	Informational messages	Clinicians expressed frustration with these purely informational messages as irrelevant to them or duplicative and therefore a source of tedious inbox work, but some did also note the importance of these messages for staying up to date with specialist care.	This was somebody who had a set of labs done yesterday. But [with a] MedStar specialist also within the team. So [the system] has forwarded [the specialist] the [results], and now I’m part of that chain and so I’ll be getting all of these [messages], but this is good to have because I would like to know it, but I can’t just delete it. (Participant 5)

### Theme 1: Workflow norms and rules

Interview subjects discussed approaches to manage the inbox by establishing clinic- or practice-wide expectations for message-related workflows. These approaches varied in the scope of other staff members involved in these workflows as well as their reported rates of success and ongoing points of friction.

#### Subtheme 1A: Team-based workflows with clinical teams (n = 10)

Medical assistants (MAs) were the clinical care team members most likely to be involved in inbox message management approaches. Most participants referenced a specific MA with whom they worked directly, and others mentioned practice-level approaches like a one-to-one matching of MAs with physicians. Some participants had implemented specific team-based workflows for certain types of clinical data (eg, laboratory results), and others had established communication expectations with other clinicians to ensure that all team members were informed of different steps in the patient outreach and/or follow-up process.

*Yeah, like for example, when I’m addressing my labs,****I send my lab results to my medical assistant****. Now if there is something that my medical assistant is getting a glitch or patient is asking more questions, she will route that message back to me.* (Participant 10)

*This patient … got rejected to do her surgery due to insurance issues. So I had my medical assistant call her insurance to see who would be in network to get this surgery done …****this is the message my medical assistant left me [to tell me] that [the MA] told [the patient].****And then that’s in my inbox. So things that I’m just looking out for, but they’ve already know that this is in process.* (Participant 4)

#### Subtheme 1B: Team-based workflows with administrative teams (n = 10)

Participants also discussed inbox management approaches that involved workflows with non-clinical administrative teams within their clinic (eg, front desk and registration staff) or external to it (eg, central call centers). These workflows tended to involve more back-and-forth communication and were often discussed as a source of friction.

*We have a****clinical pool and an administrative pool****. The administrative pool will pick up the messages that are for patients needing to get scheduled and they’ll try to put those [on the schedule]. But then sometimes that will come back, because if there’s no availability, then they send us a message [asking] “Where can we put this patient? They have to be seen for this surgery on this date.” Then I have to then go back and see where I can squeeze them in. So you know****it’s helpful, but then there’s also sometimes that we have to jump in on those same administrative tasks***. (Participant 4)

*Mainly when the call comes to our****front desk, it goes to our [administrative staff] who do not have much of the clinical knowledge.*** *And then … it comes to [the physician] and … if it is a clinical thing, I send it to my medical assistant and she calls the patient. Sometimes I pick up the phone. That’s where most of the triage is.* (Participant 10)

*Sometimes, so sometimes [my MA] will see the message and be like they’re requesting an appointment …****and [the MA will] push that to … my scheduler and then [the scheduler] will call the person to schedule****. Sometimes I will get messages [from the scheduler] because my schedule is so jampacked.* (Participant 9)

#### Subtheme 1C: Clinical scope of practice and message routing (n = 10)

Prior to this study, MedStar Health implemented a program in its primary care clinics to provide virtual nurse practitioner (NP) support to physicians. These virtual NPs have a clear scope of practice, in that they handle medication refill requests that patients send via the patient portal and provide flexible appointment availability for same-day or next-day visits that can occur virtually and do not require the primary care clinician. These clinicians also offered important care management services in some instances when patients did need to see their primary care clinician. This program, Connected Primary Care, was mentioned favorably on several occasions as a particularly valuable team-based inbox management workflow for medication refills with virtual clinical team members.

* …Connected Primary Care where now I have a group that’s refilling my medications, except for the controlled substance ones or ones that are questionable.****So that’s been like 2 weeks, it’s significantly decreased messages.***(Participant 1)

*Having the****Connected [Primary] Care program doing the prescriptions has been a life changer**** … it’s taken away so much of that load … they are able to take that extra step of not just signing off on and just get rid of it from my box, but looking back to see that actually they haven’t been seen for a year and a half or their last blood pressure was really not controlled so they need [a visit], then [the NP can make] that connection to my staff … to get in for an appointment.* (Participant 5)

#### Subtheme 1D: Workflows or habits for when to reply to messages (n = 9)

Finally, participants expressed consistently checking their message inbox throughout the clinic day, primarily to avoid reaching the end of the day with a large amount of unread messages in their inbox.

*I’m constantly looking at [my messages] very briefly during the day, so I****don’t end up at 4 o’clock with 20 messages***. (Participant 1)

*Throughout the clinic day, I would say****there’s not really a set structure, [I’m] constantly monitoring, even over the weekend**** … People could be sending urgent or emergent things that could fall through the cracks. And if I’m on vacation or I’m doing something with my family, I’m not always looking at it, but****I don’t feel comfortable letting it go … for a long period of time***. (Participant 8)

### Theme 2: Boundary setting

Interview subjects discussed different ways they implemented practices to establish boundaries to prevent inbox work from overwhelming them or becoming imbalanced across physicians their practice (eg, during vacation coverage). These practices varied primarily in terms of the subject with whom the boundary-setting practice was drawn.

#### Subtheme 2A: Boundary-setting with patients (n = 10)

Participants noted the necessity of proactively communicating with patients about expectations for the use of the portal and clinician messaging capabilities. Specifically, communication during initial visits to establish a relationship or automated text included with early portal communications typically involved setting expectations regarding turnaround time and what types of messages were and were not appropriate for asynchronous communication tools. Clinicians also commonly noted Message Center capabilities that allowed for clinicians to proactively disallow patient responses to a given message and limit back-and-forth communication or lengthy message threads.

*I have a smart text when they [first] come in … saying “Results will be in the inbox.****Give me a little bit of time to review it. Don’t put anything urgent in the inbox****.”* (Participant 11)

*I usually select “****Disable Response****.”* (Participant 1)

#### Subtheme 2B: Boundary-setting with other clinicians (n = 6)

The most common use case for establishing boundaries with other clinicians was related to covering other clinicians’ inboxes during their vacations. Participants noted varied cultural expectations across clinics as well as specific policies (official or unofficial) to facilitate shared inbox coverage without overwhelming any individual clinician.

*Like you should never leave anything in your box for somebody before you go …philosophically****our thing is before you go, don’t leave any garbage*** *for people to sort through while you’re gone.* (Participant 2)

*We’ve come into understanding****even on vacation, we check our own messages****. So we haven’t had to use that pool.* (Participant 3)

***When someone’s out the other doctors really try to make them come back to zero messages****. It is a group mission and what goes around comes around. So we all work harder and it’s a pain. But [the goal is] to come back to no messages.* (Participant 1)

#### Subtheme 2C: Boundary-setting with oneself (n = 7)

Finally, participants expressed boundaries that they set with themselves to not allow their inbox work to overwhelm them. Some of these boundaries involved specific periods during the week that the clinician specifically did not reply to messages or check their inbox, while others expressed a desire to set these boundaries or instances when these boundaries conflicted with other individual and organizational goals for responsive, high-quality care.

*I****definitely don’t log on on the weekends, I work Monday through Friday and that’s it****. If I’m on vacation, I would log in, you know, maybe a couple of times just [so] I won’t come back to 1000 messages, you know?* (Participant 3)

*[I’ll instruct some patients to] just send me a message because you know, I know how to access the pool. I know how to do this so****I know part of that is my mistake****, but it’s better than the patients safety being impacted.* (Participant 5)

*Yeah, it’s like finding that fine line … I want to have these patients that are satisfied with my care … and it’s refreshing when I get my reviews … in our finance meeting we get the patient comments. And so I see it reflected in that, but****[I’m] also trying to set these boundaries for my peace of mind.****It’s like that constant struggle of trying to do that … so that’s a hard part.* (Participant 4)

### Theme 3: Message classification heuristics

During inbox observations, subjects indicated 3 key classes of messages that helped inform how they would engage with a given message. Classes were primarily distinguished by how long it would take the clinician to complete whatever the message required from them, which in some cases was nothing and in others was considerable follow-up. Clinicians reported prioritizing messages that could be completed or dealt with quickly over those taking more time or cognitive energy.

#### Subtheme 3A: Messages requiring follow-up or additional chart review (n = 8)

Relatively few messages fell into the category of messages that necessitated further follow-up or chart review from the clinician. While these messages were relatively rare, clinicians indicated that these messages required a disproportionate amount of time and cognitive energy, and often involved care coordination tasks.

*That one would probably be something I would say like, OK,****I need to look into it further cause I need to look at her****chart. I need to figure out what other kind of medical conditions she has … I was going to look into that, but just given time, I’m gonna probably skip ahead right now and come back to that one.* (Participant 5)

*They’re always there, always things that take more time, like one of the messages in my inbox right now is someone who gave me an update on her health and wants to know if I know what geriatric specialist for her mom.****So that’s been sitting there for six days because I’m thinking about it****. I’ll put out some feelers.* (Participant 1)

#### Subtheme 3B: Messages requiring minimal work (n = 9)

A large volume of messages fell into the category of messages that could be quickly managed via immediate deletion or acknowledgement.

*So this is something I can address really quickly. So I’ll go in her actual chart to see if I can see a letter. Then I’ll just. I’ll do it. A lot of it has to do with like speed, like how quickly I can address the message. So****I try to do the, you know, quick, quick, quick ones first***. (Participant 3)

*So quick [messages like] “I need an order for this.” I’ll put that in real quick. [Or a message asking] “Do you think the patient needs to be seen for this issue?” I reply back, “Yes, schedule the patient for an urgent appointment.” It’s****a quick yes, no or a couple clicks***. (Participant 4)

***Prescriptions are fairly quick****, so that I’ll try and knock them out before I’m done [for the day]* (Participant 5)

#### Subtheme 3C: Informational messages (n = 7)

The last set of messages comprised those that were not distinguished by the time it would take to manage them but rather by the fact that they consisted purely of informational updates that the clinician did not need to act on. Clinicians expressed frustration with these messages as irrelevant to them or duplicative and therefore a source of tedious inbox work, but some did also note the importance of these messages for staying up to date with specialist care.

*The “sign and review” inbox [section] is generally just faxes from doctors****. Imaging reports are always duplicative.*** *I’ve already contacted [the informatics team] and they said there’s nothing they can do about that for now because they’re required to just scan all the faxes.* (Participant 6)

*These are just messages from outside hospitals … I actually saw [this] message yesterday that this patient was admitted to the ER and for some reason [a non-MedStar site], when they send us messages for each ER visit, they’ll send like 6 messages …****They send like 2-3 [duplicate] copies of the same message.*** *I’m gonna get a discharge notice, so I’ll save that to the chart and then I’m done and then I can see the next [message from this site]. See this is the same exact discharge notification.* (Participant 7)

*This was somebody who had a set of labs done yesterday. But [with a] MedStar specialist also within the team. So [the system] has forwarded [the specialist] the [results], and now I’m part of that chain and so I’ll be getting all of these [messages], but****this is good to have because I would like to know it, but I can’t just delete it.*** (Participant 5)

## Discussion

We conducted semi-structured interviews with 11 primary care clinicians to discuss their strategies for inbox message prioritization and overall message management. In general, clinicians did not report prioritizing messages, primarily because of time constraints. As a result, our initial hypothesis that prioritization could be observed from current practice and subsequently systematized into algorithmic tools was not supported. These findings fit with recent literature highlighting time constraints in primary care generally.^16–18^ Clinicians in our study highlighted several structural reasons why prioritization was not a priority, chiefly that all messages had to be handled eventually and they had no dedicated time to organize messages (other than time they made for themselves by coming in early, staying late, or working on weekends). As a result, any time spent prioritizing only added to the overall time the clinician spent doing inbox work, rather than facilitating inbox workflows that could potentially save time.

The inbox management workflows that clinicians did note as important sources of practice efficiency nearly universally involved MAs, who served numerous roles as care coordinators and patient communication liaisons responsible for discussing scheduling, test results, and specialist referrals with patients directly. Inbox workflows with MAs were typically specific to the individual clinician and relied on co-location and strongly enforced workflow norms for success. Other inbox management approaches that involved administrative teams (eg, front desk or call center staff) were more difficult to establish with as much consistency, largely because these teams were highly varied in their own workflows and in many cases were not co-located with the clinic staff. As a result, these administrative workflows did not always fit well with the existing or preferred workflows for our clinician participants. Organizations aiming to improve collaborative workflows between decentralized, individual clinics and centralized or shared administrative teams—especially call centers—should consider developing strong standardized processes and workflows for these centralized teams to ensure that clinic staff and clinicians are not forced to adapt to different workflows originating from the same source.

Our third key finding focuses on how clinicians implemented different forms of boundary-setting for patients, other clinicians, and themselves. Clinicians cited pre-emptive communication strategies with patients to establish expectations for how and when to use portal messaging most effectively, as well as to discourage inappropriate portal use (eg, for emergencies). Boundary-setting with other clinicians primarily involved clinic-level expectations for inbox coverage while clinicians were on vacation,^19^ specifically that the vacationing clinician should not leave messages in his or her inbox from prior to their time off for other clinicians to manage. In exchange for this pre-vacation inbox management, some clinics had implemented strong norms to ensure that vacationing clinicians came back to no inbox backlog; others had expectations that clinicians continue to manage their own inbox while on vacation. Future research should explore patients’ understanding of appropriate portal messaging use and the efficacy of pre-emptive communication with patients regarding appropriate portal use, as well as the wellbeing costs of different approaches to vacation coverage (for both vacationing clinicians and those tasked with inbox coverage in their absence). Finally, clinicians expressed a desire to set boundaries to protect themselves from overwhelming inbox work, acknowledging that “drawing a line” would be protective to their wellbeing. However, clinicians noted that implementing this in practice was challenging because of pressures to be responsive to patients, especially as patients specifically cited responsiveness as a positive aspect of their care experience. Notably, most subjects who mentioned these self-imposed boundaries illustrated a belief that establishing sustainable work patterns for managing the inbox fell entirely on themselves, with little indication of organizational supports to help implement or maintain these boundaries.

Our study has several limitations to note. First, we conducted our study at a single organization, MedStar Health, and although our clinician participants represented varied primary care practices, our findings may not generalize outside of the MedStar Health context. Second, our convenience and snowball recruitment methods yielded a sample of participants that were predominantly female, so our findings may be more representative of the female clinician experience than the male experience. Past research has shown a gender gap in EHR use time, support staff, and inbox burden, so our findings should be interpreted with our specific sample in mind.^20–23^ Finally, our results necessarily present a subset of the universe of themes and subthemes identified by the research team. While other themes and subthemes were present, they did not occur with sufficient frequency to be included in our results.

## Conclusion

In semi-structured interviews and observations with 11 primary care clinicians, we found that time constraints limited clinicians’ ability to prioritize inbox messages. The most common themes related to inbox management were the establishment of collaborative workflows with clinical and administrative teams, especially with MAs. Clinicians also noted different strategies for setting boundaries with other parties and for themselves, though the structure and implementation of these boundaries varied across clinicians and practices. Our results can inform organizations aiming to reduce clinician inbox burden and improve inbox management workflows as well as support future research exploring the efficacy of different inbox management approaches via quantitative evaluation studies.

## Supplementary Material

ooae135_Supplementary_Data

## Data Availability

The data underlying this article cannot be shared publicly to protect the privacy of individuals that participated in the study. De-identified data may be shared on reasonable request to the corresponding author.
