# Supplementary material for: Inbox message prioritization and management approaches in primary care
Source: JAMIA Open. 2024 Nov 11;7(4):ooae135. doi: 10.1093/jamiaopen/ooae135 (PMC11552621; doi:10.1093/jamiaopen/ooae135)
Supplement: ooae135_Supplementary_Data [file ooae135_supplementary_data.docx]

**Appendix Table. Summary of Secondary Themes and Subthemes**

| **Theme** | **Subtheme** | **Summary of Findings** | **Illustrative Quote** |
| --- | --- | --- | --- |
| Patient characteristics | Patient known to provider | Whether a patient was well known to interview subjects influenced how the subject responded to their message. Knowing the patient occasionally factored into decisions about whether to address inquiries via message or appointment. | ...Sometimes when I get those hospital messages, if it's a name that I recognize that [they are] at high risk for readmission, I will actually forward it to my front desk to make sure they have a hospital follow up... (Subject 6) |
| Issues affecting patients | Scheduling or appointment availability constraints | Interviewees reported that they sometimes fulfilled patient requests via message that they normally would have preferred to handle in an appointment if the patient had tried and failed to be seen in a timely manner. | ...If I had had a same day appointment available, I probably would have made them do a virtual appointment with me for this refill and lab order. But because I have [no appointments] available, I'm just going to put it in... (Subject 6) |
|  | Requests made via messages | Some interviewees expressed concern that patients took advantage of the message portal and used it like an “advice line.” They noted that clinical decisions should not be made via the portal. | Things where I need to make clinical decisions, they need to be an appointment (Subject 4) |
| Use of efficiency tools | Message drafting tools | Many interviewees highlighted the use of dictation tools in their messaging workflows. In the most efficient cases, these were combined with templated text like a standard lab interpretation or disease management plan. | “… I dictate everything. I’m like, a mad dictator. I never type because these kinds of things you can get through way, way faster if you're dictating.” (Subject 2) |
